# Short learning curve in transition from laparoscopic to robotic-assisted rectal cancer surgery: a prospective study from a Finnish Tertiary Referral Centre

**DOI:** 10.1007/s11701-023-01626-7

**Published:** 2023-07-08

**Authors:** Charlotta S. J. Kolehmainen, Mika T. Ukkonen, Timo Tomminen, Ilona M. Helavirta, Johanna M. Laukkarinen, Marja Hyöty, Sannamari Kotaluoto

**Affiliations:** 1 Faculty of Medicine and Health Technology, Tampere University, Kauppi Campus, Arvo Building, Arvo Ylpön katu 34, 33520 Tampere, Finland; 2 Department of Gastroenterology and Alimentary Tract Surgery, Tampere University Hospital, Tampere, Finland

**Keywords:** Robotic surgery, Rectal cancer surgery, Learning curve, Surgical training

## Abstract

The narrow pelvis causes special challenges in surgery, and robotic-assisted surgery has been proven beneficial in these circumstances. While robotic surgery has some specific advantages in rectal cancer surgery, there is still limited evidence of the learning curve of the technique involved. The aim here was to study the transition from laparoscopic to robotic-assisted surgery among experienced laparoscopic surgeons. The data for this study were collected from a prospectively compiled register that includes patients operated on by the Da Vinci Xi robot in Tampere University Hospital. Each consecutive rectal cancer patient was included. The information on the surgical and oncological outcomes was analysed. The learning curve was assessed using cumulative sum (CUSUM) analysis. CUSUM already demonstrated an overall positively sloped curve at the beginning of the study, with neither the conversion rate nor morbidity reaching unacceptable thresholds. Conversions (4%) and postoperative complications (Clavien–Dindo III–IV 15%, no intraoperative complications) were rare. One patient died within one month and the death was not procedure-associated. While surgical and oncological outcomes were similar among all surgeons, the console times showed a decreasing trend and were shorter among those with more experience in laparoscopic rectal cancer surgery. Robotic-assisted rectal cancer surgery can be safely adapted by experienced laparoscopic colorectal surgeons.

## Introduction

Robotic-assisted surgery is an advanced surgical method with special benefits in pelvic surgery [1, 2]. As minimal invasive surgery has become a standard in colon cancer surgery, rectal surgery still is still on the journey due to the limitations of the laparoscopic approach in the lower pelvis [3, 4]. Robotic surgery seems more promising in rectal surgery than the conventional laparoscopic approach, and thus some units performing rectal surgery have transferred from open surgery straight to robotic surgery [5]. Robotic surgery has offered a new way of operating expanding the population of colorectal cancer patients eligible for minimally invasive surgery.

While the robotic-assisted surgery has some specific advantages, only few studies have reported the technique-associated learning curve among rectal cancer patients. A cumulative sum (CUSUM) analysis can be used to assess surgical performance and learning curve when acquiring new surgical skills [6–8]. Here we study the learning curve associated with the shift from laparoscopic to robotic rectal cancer surgery among experienced laparoscopic and open surgeons.

## Materials and methods

All colorectal cancer patients operated with DaVinci Xi robot (Intuitive Surgical, Inc) between October 2017 and September 2019 were included in the prospective study register. Of 201 patients, 139 had rectal cancer and were included in this study.

The prospectively collected register on which this study was based includes information on patient demographics, preoperative treatment, operative and pathological characteristics, operation time, adjuvant therapy, 30-day morbidity and mortality. The robotic console time (time from finished docking to the end of console work), docking time (time taken to position the robot and mount the robotic arms) and total operative time were gathered. Rectal cancer was defined as histologically proven carcinoma within 18 cm of the anal verge in MRI (magnetic resonance imaging) [9]. Tumour height was defined as low (0–6 cm), middle (6–11 cm) or high (12–18 cm), measured from the anal verge in pre-operative MRI as defined in the Finnish Colorectal Cancer Guidelines [9]. Complications were defined and graded according to the Clavien–Dindo classification of surgical complications [10]. Thirty-day mortality and morbidity were registered.

Neo-adjuvant therapy was designed for each patient, as in standard care, by a multidisciplinary team. Short course radiotherapy (SRT) consisted of 5 × 5 Gy with immediate surgery within seven days, long course chemo-radiotherapy (LCRT) consisted of 50.4 Gy to tumour area and 45 Gy to lymph node area divide into to 1.8 Gy daily, using fluoropyrimidin (Cabesitabine^®^) as a radiotherapy enhancer. Short course radiotherapy with interval (SRT-I) consisted of 5 × 5 Gy and a waiting period of 6–10 weeks before surgery was used for fragile patients who were not responsive to chemo-radiotherapy. These treatments are the appropriate approaches for rectal cancer according to the Finnish colorectal cancer guidelines [9].

Four senior surgeons performed the operations. All the surgeons in this study were skilled in the laparoscopic and open technique, and two of the surgeons had previous experience of 20 operations on the earlier model of DaVinci S (Intuitive Surgical, Inc). Two of the surgeons had experience with over 200 cases in both open and laparoscopic rectal cancer, two surgeons had experience of approximately 50 cases in both. If console surgeon was less experienced, they were assisted by the more experienced surgeon After robotic surgery was introduced, there was no patient selection between laparoscopic and robotic approach within the limit of the resources available (two operating theatre days per week). All patients suitable for minimally invasive surgery were operated on with the robot. After gaining experience, high rectal cancers were selected for conventional laparoscopy if the robot resource was not available. In our experience robotic surgery has advantages, especially when operating on middle-level and low tumours. Selection between surgeons was usually done according to the experience of the surgeon. This selection might have affected the results between surgeons, thus the ideal time for surgery was defined for each surgeon individually.

### The CUSUM analysis

The CUSUM method was used for learning curve analyses and conducted separately for each surgeon. The method is basically a graphical representation of the trend in outcomes of a series of consecutive procedures performed over time. The CUSUM score is plotted on the y-axis against the number of operations on the x-axis. For each success, the number (s) is subtracted from the previous CUSUM score. For each failure, the number (1 − s) is added to the previous CUSUM score. Horizontal lines are plotted at regular intervals on the y-axis, defining h_1_ (unacceptable boundary lines) and h_0_ (acceptable boundary lines). When an individual is performing at an unacceptable level, the CUSUM curve slopes upward and will eventually cross an unacceptable boundary line. The CUSUM formulae used in this study are described in the Appendix.

The CUSUM analyses were done on the conversion, intraoperative complication and leakage rate individually. While the failure rate of experienced surgeons is well described in the literature, the corresponding data for learners is scarce. We consider causes for overall morbidity are multifactorial, and therefore focus solely on intraoperative complications (bleeding, perforation or other unexpected incident leading to conversion or additional procedures during the operation) and leakage (radiologically or endoscopically diagnosed leakage treated conservatively or operatively). We are interested specifically in when the performance becomes acceptable. The probability of failures is shown in Table 1.

**Table 1:** Probability of failures used in CUSUM analysis

	Conversion	Complication^a^	Overall
Acceptable failure rate (p_0_)	4%	10%	14%
Unacceptable failure rate (p_1_)	8%	15%	23%
Decrement with success (s)	0.06	0.12	0.18
Increment with failure (1 − s)	0.94	0.88	0.82

^a^Intraoperative complication or postoperative anastomotic leakage

### Statistical analysis

All statistical analyses were performed using SPSS Statistics version 22 for Windows (IBM Corp, Armonk, NY). Non-categorical data is presented as median with the inter-quartile range (IQR) or range. Chi squared test or Fisher’s exact (when expected cells value was 5 or lower) tests were performed to compare categorical variables and Mann–Whitney or Student’s t test to compare continuous variables. Linear regression model was used to analyse the correlation between numbers and durations of operations. All the tests used were two-tailed. Statistical significance was set at a p value of < 0.05.

### Ethical aspects

The study was conducted according to the Helsinki Declaration and institutional review board approval was obtained (R19517S).

## Results

During the study period four surgeons operated on a total of 139 patients, of whom 50 (36%) were female and 89 (64%) were male. The median age of patients was 70 (48–89) years and body mass index was 26 (16–37) kg/m^2^. The median for lymph nodes harvested during surgery was 22 (7–79). CRM was positive in 9.4% of the operations. Anterior resection was performed on 72 (52%) of the patients. Patient and disease specific characteristics are presented in more detail in Table 2.

**Table 2:** Patient and disease-specific characteristics (n = 139)

Variable	
Age (median)	70 (48–89) years
Sex, female	50 (36%)
Body mass index (kg/m^2^, median)	26 (16–37)
ASA score (2 missing)	
1	6 (4.3%)
2	68 (49%)
3	61 (44%)
4	2 (1.4%)
Preoperative albumin (median)	36 (27–44)
Preoperative Hb (g/l, median)	133 (86–180)
Postoperative Hb (g/l, median)	107 (75–139)
Preoperative treatment	
None	32 (23%)
Short course radiotherapy	48 (34,5%)
Long course chemoradiotherapy	48 (34,5%)
Short course RT with interval	11 (8%)
Stage	
CR^a^	5 (4%)
I	43 (31%)
II	27 (19%)
III	57 (41%)
IIII	7 (5%)
Harvested lymph nodes (median)	22 (7–79)
Distal marginal cm (median)	3 (0–10)
CRM mm (median)	5.5 (0–30)
CRM positive	13/139 (9.4%)
Anterior resection	72 (52%)
Location of tumor	
Low (0–6^b^)	58/139 (42%)
Mid (7–11^b^)	62/139 (44%)
High (12–18^b^)	19/139 (14%)

^a^Pathological complete response after neoadjuvant therapy

^b^Distance from anal verge (cm)

The operative outcomes are presented in Table 3. The conversion rate was 4% (6/139), of which one operation was converted into a laparoscopic approach and five into open surgery. Reasons for conversion to open surgery was perforating growth of the tumour, severe adherents, and bleeding and extremely narrow pelvis. In one case after finding compromised margin, and thus the change of surgical approach, the operators’ choice was to continue preparation laparoscopically and not to dock again. One in four patients (35/139) suffered from postoperative complications (15% C-D III–IV). There were no intraoperative complications. Only two patients (3%) had anastomotic leakage. Median blood loss during the surgery was 175 ml (IQR 50–260 ml). Only one patient (1%) died within a month of the surgery, and the cause of death was pneumonia.

**Table 3:** Postoperative outcomes (n = 139)

Variable	
Intraoperative bleeding (ml, median, IQR)	175 (50–260)
Conversion	
Open surgery	5 (4%)
Laparoscopy	1 (1%)
Complications	35 (25%)
C-D* 1 or 2	14 (10%)
C-D* 3 or 4	21 (15%)
Intraoperative complications	0
Anastomotic leakage	2 (3%)
30-day postoperative mortality	1 (1%)

^a^Clavien–Dindo classification of postoperative complications

**Fig. 1:**
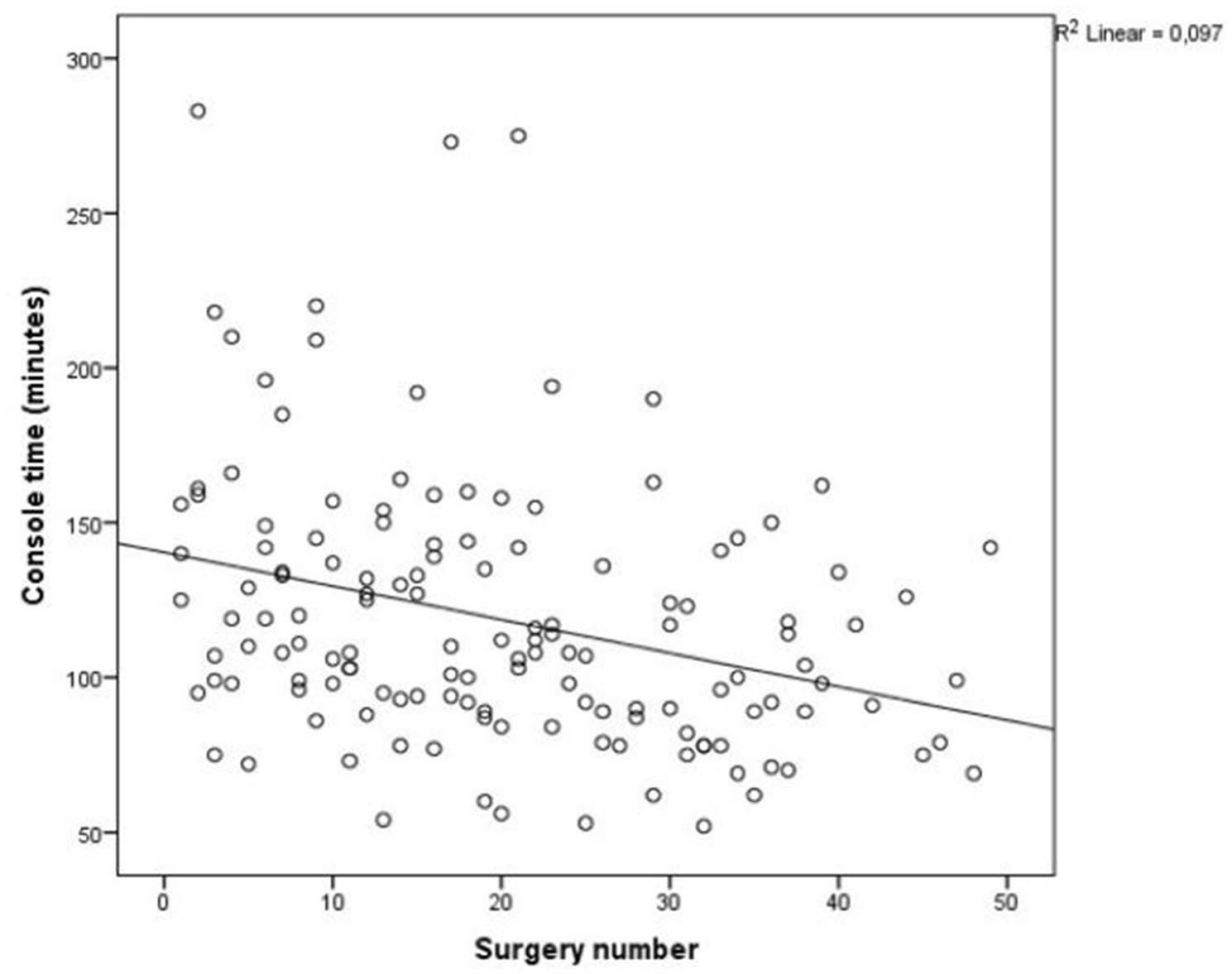
Scatterplot of the linear regression model between operation time (console time) and number of procedures (p < 0.001) showing decreasing trend in operation length against number of surgeries performed

A graph with console time plotted against surgery number is shown in Fig. 1. As seen, console times show a decreasing trend over number of procedures (R^2^ = 0.097). The median console time when surgeons had performed ≤ 20 robotic-assisted surgeries was 123 (range 96–151) min and 100 (79–123) min when surgeons had performed over 20 surgeries (p = 0.002). Other postoperative outcomes, i.e., postoperative morbidity, mortality, rate of readmissions and length of hospital stay remained similar, as shown in Table 4. When surgeons with experience of over 200 rectal cancer resections (open or laparoscopic) were compared to surgeons with experience of 50 resections, both the console time (100 [IQR 79–124] vs. 125 [96–155] min, p = 0.002) and number of lymph nodes examined (26 [12–69] vs. 20 [7–79], p = 0.017) were better, as shown in Table 5.

**Table 4:** Postoperative outcomes according to number of procedures

	≤ 20 operations	> 20 operations	p value
Console time (min, IQR)	123 (96–151)	100 (79–123)	0.002
Conversion	3/80 (3.8%)	3/69 (4.3%)	0.587
Lymph nodes examined (n, range)	20 (7–79)	25 (12–71)	0.017
Share with < 12 lymph nodes	5/70 (6.7%)	0/65 (0.0%)	0.061
Intraoperative bleeding (ml, IQR)	150 (50–272)	150 (90–250)	0.998
Leakage^a^	1/80 (1.3%)	2/69 (2.9%)	0.596
Length of hospital stay (days, range)	8 (2–40)	5 (2–20)	0.085
Readmission to hospital	7/80 (8.8%)	11/69 (16%)	0.179
Morbidity^b^	17/80 (21%)	18/69 (26%)	0.487
Mortality	1/80 (1.3%)	0/69 (0.0%)	0.537

^a^Radiologically or endoscopically confirmed leakage

^b^C-D 1–4

**Table 5:** Comparison between two surgeons with experience of over 200 cases in open or laparoscopic rectal cancer resections and two surgeons with experience of over 50 cases of both

	More experienced surgeons	Less experienced surgeons	p value
Console time (min, IQR)	100 (79–124)	125 (96–155)	0.002
Conversion	5/87 (5.7%)	1/62 (1.6%)	0.401
Lymph nodes examined (n, range)	26 (12–69)	20 (7–79)	0.017
Share with < 12 lymph nodes	3/82 (3.7%)	2/58 (3.4%)	0.660
Intraoperative bleeding (ml, IQR)	150 (60–250)	180 (100–280)	0.998
Leakage	2/87 (2.3%)	1/62 (1.6%)	0.625
Length of hospital stay (days, range)	5 (2–26)	5 (3–40)	0.540
Readmission to hospital	12/87 (14%)	6/62 (9.7%)	0.447
Morbidity	21/87 (24%)	14/62 (23%)	0.825
Mortality	0/87 (0.0%)	1/62 (1.6%)	0.416

**Fig. 2:**
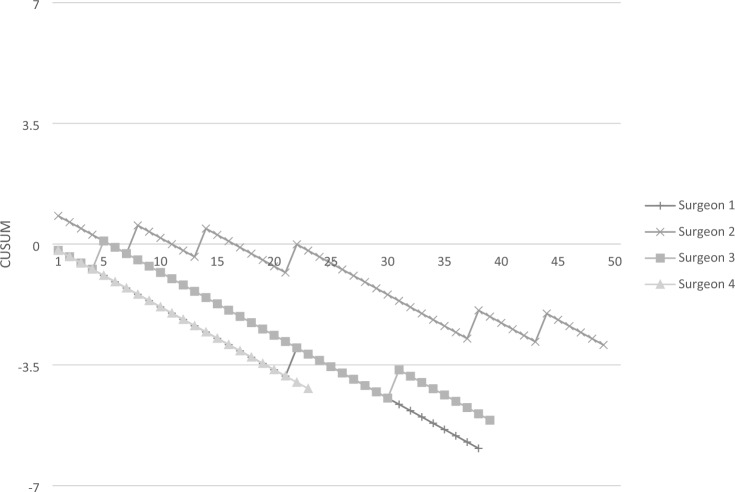
Cumulative failure graph demonstrating acceptable performance since the introduction of the new technology. Failed attempts are indicated by the upward deviations in the plot, while success is indicated by downward deviations

The CUSUM analysis is shown in Fig. 2. CUSUM already showed an overall positively sloped curve at the beginning of the study. Neither the conversion rate nor the rate of intraoperative morbidity or leakage approached the acceptable threshold of 14%.

## Discussion and conclusion

Earlier studies suggest that robotic rectal cancer could have a relatively short learning curve if a surgeon is competent in rectal surgery and has experience in laparoscopic surgery [6, 11]. In our study we were able to confirm that experienced colorectal surgeons adopted the new technique with a short learning curve and adequate surgical and oncological outcome.

Outcome of surgery was immediately on an acceptable level as a short oncological outcome. The number of conversions was low (4%) and remained low during the study for all surgeons compared to earlier results in rectal cancer surgery, both laparoscopic (12–29%) and robotic (5%) [3, 11, 12]. Complications occurred in 25% of patients (Clavien–Dindo I–IV) compared to 27–33% in earlier studies of minimally invasive rectal cancer surgery [5, 12]. Earlier studies have reported 5% positive CRM and 16 harvested mean lymph nodes compared to 9% and 22 respectively in our study [12]. The quality of TME (total mesorectal excision) is only estimated by surgeons themselves in our institute and was not considered a reliable measurement of quality of surgery. In our opinion positive CRM, involvement of tumour in the circumferential margin, is rather a reflection of tumour invasiveness and should be evaluated with the stage and quality of TME. Thus we chose to analyse only harvested lymph nodes, which is a measure of surgery, in addition to quality of pathology. LCRT affects the lymph node count, while no perfect measure for short oncological outcome is available. A failure rate of less than 12 nodes was chosen following the European and national guidelines [9, 14].

The learning curve analyses in our study showed a consistent reduction in operation time for all four surgeons. The surgeons with most experience in open and laparoscopic rectal cancer surgery had the shortest console times and most lymph nodes harvested compared to surgeons with less experience. In this study we were not able to show a cut-off value for acceptable level in operating time. On the contrary, defining optimal time for surgery is challenging due to differences in patients and tumours. Moreover, more difficult cases were operated on by more experienced surgeons.

There are some limitations in our study. The number of patients was relatively small. We lack quality grading of the TME in specimens by the pathologist. Thus the evaluation of short oncological outcome may be discussed. On the other hand the results are comparable to those of cohort studies of robotic and laparoscopic rectal cancer surgery. The strength of this study is that the results are real life. All data was collected prospectively in a robotic rectal surgery register. The operations, education for robotic surgery and selection of early cases was carried out as planned to build up a robotic rectal surgery team without compromising patient safety or surgical outcome. This also implies that the results cannot be generalized to any surgeon embarking on robotic surgery. We were nevertheless able to show that a colorectal surgeon with enough experience to learn a new surgical technique, has a relatively short learning curve for a robotic approach with Da Vinci Xi. In addition, standardized robotic education following the Intuitive learning path is mandatory for starting robotic surgery.

## Conclusion

Robotic surgery can be introduced into rectal cancer surgery safely with a short learning curve in dedicated colorectal centres.

## Data Availability

The data that support the findings of this study are available on request from the corresponding author, CK. The data are not publicly available due to restrictions containing information that could compromise the privacy of research participants.

## Appendix

Formulae according to Bolsin and Colson [13]:

$${\text{a}} = \ln \{ (1 - \upbeta )/\upalpha \}$$

$${\text{b}} = \ln \{ (1 - \upalpha )/\upbeta \}$$

$${\text{P}} = \ln ({\text{P}}1/{\text{P}}0)$$

$${\text{Q}} = \ln \{ (1\, - \,{\text{P}}0)/(1\, - \,{\text{P}}1)\}$$

The decrement and increment of each success (s) or failure (1 − s):

$${\text{s}} = {\text{Q}}/\left( {{\text{P}} + {\text{Q}}} \right)$$

The spacing between unacceptable (h_0_) and acceptable (h_1_) boundary lines:

$${\text{h}}_{0} = {\text{p}}/\left( {{\text{P}}\, + \,{\text{Q}}} \right)$$

$${\text{h}}_{{1}} = {\text{a}}/({\text{P}}\, + \,{\text{Q}})$$
